# Microglia-astrocyte crosstalk following ischemic stroke

**DOI:** 10.1186/s13041-025-01244-4

**Published:** 2025-10-01

**Authors:** Shangsong Yang, Yuxiong Chen, Jialin Tang, Yicheng Cui, Wei Wei, Zhongnan Hao, Zhipeng Xiao, Yongli Pan, Qinyuan Tian, Wenqiang Xin, Meihua Li

**Affiliations:** 1 Jiangxi Key Laboratory of Neurological Diseases, Department of Neurosurgery, The First Affiliated Hospital, Jiangxi Medical College, Nanchang University, Nanchang, Jiangxi China; 2 Queen Mary School, Nanchang University, Xuefu Avenue, Nanchang, Jiangxi China; 3 Department of Transplantation, The First Affiliated Hospital, Jiangxi Medical College, Nanchang University, No. 17 Yongwaizheng Street, Nanchang, 330006 Jiangxi China; 4 Department of Neurology, the Affiliated Hospital of Southwest Jiaotong University & The Third People’s Hospital of Chengdu, Chengdu, 610031 Sichuan PR China; 5 Department of Neurology, University of Göttingen Medical School, Göttingen, Germany; 6 Department of Neurology, Shandong Provincial Hospital, Shandong First Medical University, Jinan, Shandong P. R. China

**Keywords:** Microglia, Astrocyte, Crosstalk, Ischemic stroke, Inflammation

## Abstract

Ischemic stroke, the most prevalent form of stroke, severely impacts human health due to its high incidence, disability, and mortality rates. The complex pathological response to ischemic stroke involves the interplay of various cells and tissues. Among these, astrocytes and microglia, as essential components of nervous system, play significant roles in the pathological processes of ischemic stroke. In addition to their individual functions, an increasing number of studies have revealed that the interaction between astrocytes and microglia is crucial following ischemic stroke. It integrates current research reports to examine and clarify the effects of interaction between the microglia and astrocytes on the nervous system after ischemic stroke, aiming to provide new insights and approaches for future academic research and disease treatment.

## Introduction

Stroke, a complex cerebrovascular condition, is a leading cause to mortality and disability across the world [1]. Latest data from the World Health Organization indicate that ischemic stroke is the second most common cause of death globally [2]. In the central nervous system (CNS), astrocytes and microglia, which are crucial cellular components, become activated following an ischemic stroke and play distinct pathological roles during its various phases, exerting substantial influences on disease’s pathological processes. These two types of glial cells have a dualistic function, manifesting both protective and detrimental effects on the outcome of the stroke. These two types of glial cells exhibit a dual role in the progression of stroke: they can both exert protective effects and cause detrimental impacts. For instance, during the acute phase of stroke, microglia can mitigate inflammation, but they subsequently shift towards a pro-inflammatory phenotype [3, 4]. Meanwhile, during the acute phase, astrocytes are rapidly activated, proliferate, and secrete GFAP to restrict inflammatory, and in the subacute phase, they facilitate the repair of damaged tissue [5]. Additionally, microglia-astrocytes interaction also plays a double-edged sword role in ischemic stroke. On the one hand, this interaction can exacerbate damage by releasing pro-inflammatory factors and detrimental cytokines. On the other hand, it can also trigger the secretion of anti-inflammatory factors and protective cytokines, which help to mitigate the inflammatory response and safeguard the nervous system and neurons [6]. This review investigates the diverse roles of these two types of glial cells in ischemic stroke and provides a detailed analysis of how their interactions impact ischemic stroke. It aims to a more profound comprehension of the responses occurring in the context of ischemic stroke, elucidate the underlying principles and offer new perspectives and direction for clinical applications and scientific research.

## Dynamic microglia activation after ischemic stroke

This review summarizes the existing research findings and examines the activation of microglia, the immune cells resident in the CNS, in context of ischemic stroke. It particularly focuses on the activation process and phenotypic transformation of microglia following ischemic stroke, and provides a detailed analysis of the impact of these changes on the nervous system.

To gain a comprehensive understanding of the roles that microglia play in different stages of ischemic stroke, researchers conducted a thorough literature search on databases such as PubMed and CNKI for studies published from 2016 to the present. The search employed keywords including “Microglia,” “Activation,” “Ischemic stroke,” “Microglia spectrum,” and “Inflammation.” Additionally, researchers manually reviewed reference lists to identify other relevant studies. This iterative process continued until no new relevant studies were identified.

As the CNS’s indigenous immune cells, microglia are triggered to activate and migrate towards the lesion site following an ischemic stroke [7, 8]. After ischemic stroke occurs, brain tissue cells are damaged, and the integrity and function of the BBB are compromised [9]. This damage triggers the release of a large number of inflammatory mediators and cytotoxic factors, which act as external stimuli to activate microglia [10]. In addition to external stimuli, stroke also disrupts the balance of the nervous system, and this internal imbalance can trigger the activation of microglia through intrinsic mechanisms [11, 12]. However, after ischemic stroke, the damage and death of neurons lead to a significant decrease in the expression of CD200 and CX3CL1, disrupting this balance and thereby causing the activation of microglia [11–13]. Within microglia, the pro-inflammatory factor IRF5 and the anti-inflammatory factor IRF4 antagonize each other to maintain their resting state [14]. After ischemic stroke, this balance is disrupted, with increased expression of IRF5 and relatively decreased expression of IRF4, leading to the activation of microglia and their polarization towards a pro-inflammatory phenotype [14].

Conventionally, it has been believed that upon activation, microglia can differentiate into two distinct phenotypes: Microglia-1 type (M1) and Microglia-2 type (M2) [3]. M1 microglia release cytotoxic cytokines that can induce brain necrosis. Studies using the middle cerebral artery occlusion (MCAO) model have shown that NF-κB translocates from the cytoplasm to nucleus [15–17]. NF-κB triggers the production of pro-inflammatory cytokines, including interleukin-1β (IL-1β), IL-6, tumor necrosis factor-α (TNF-α), and inducible Nitric Oxide Synthase (iNOS) [15]. These cytokines promote inflammation and brain edema, resulting in a poor prognosis. In contrast, M2 microglia release anti-inflammatory cytokines and engulf injured tissue and debris. For instance, the secretion of IL-4 has been shown to reduce the size of infarcts post-ischemic stroke and enhance long-term functional recovery [4, 15]. Additionally, the secretion of Chitinase 3-like protein 3 (Ym1/2), IL-10, and transforming growth factor-β (TGF-β) has been shown to promote angiogenesis, reduce blood-brain barrier (BBB) permeability, and improve outcomes following a stroke. Furthermore, elevated levels of TGF-α have been established to enhance the proliferation and neuronal differentiation of neural stem cells following ischemic stroke [18].

**Fig. 1:**
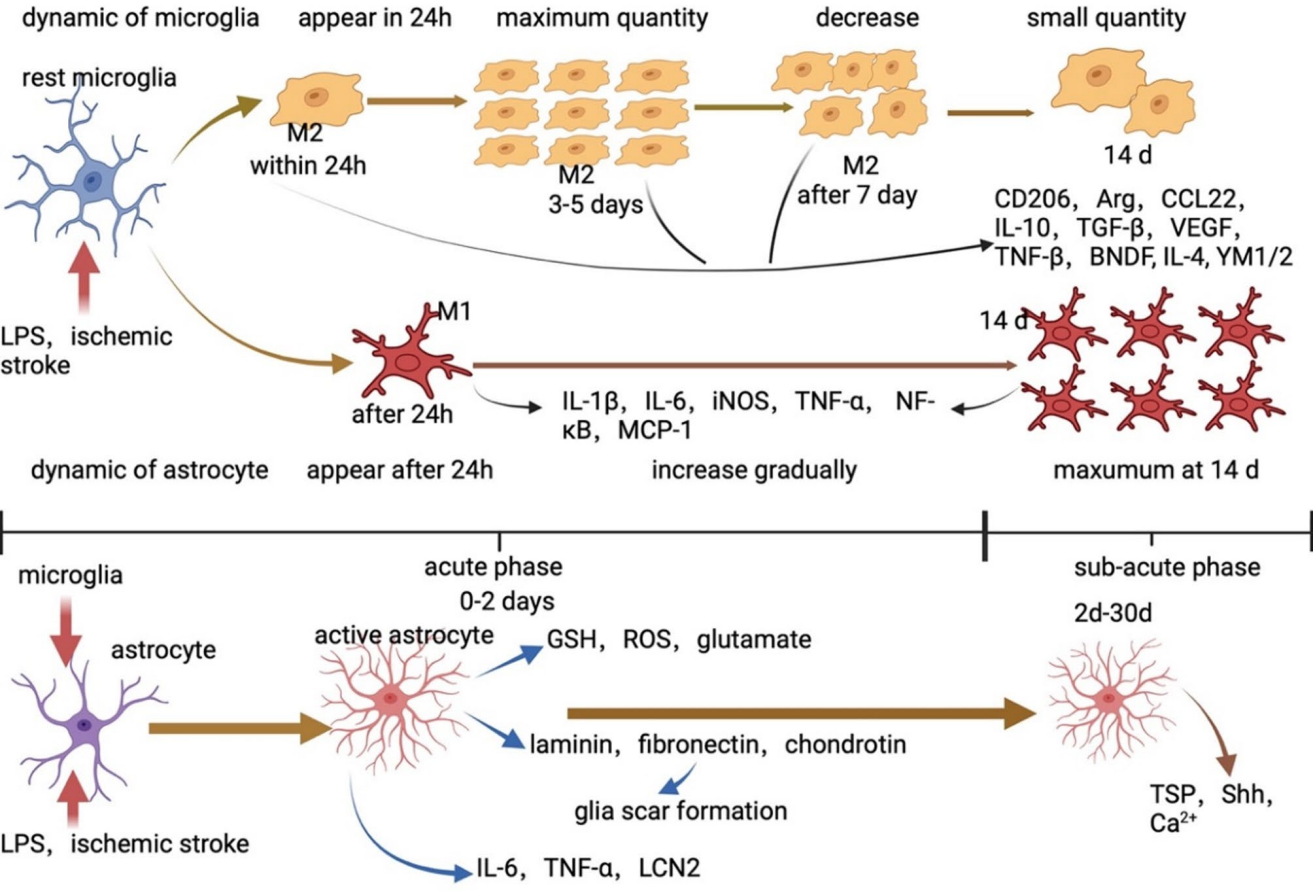
The dynamic changes of astrocytes and microglia following ischemic stroke and their functional roles. In the context of ischemic stroke or LPS stimulation of glial cells, the period from 1 to 7 days is considered the acute phase, and after 7 days, it is considered the subacute phase. Under the stimulation of LPS or ischemic stroke, resting microglia transition to an activated state. M2-microglia begin to appear within 24 h and the number of microglia peaks 3 to 5 days into the inflammation. After one week, as the inflammation enters the subacute phase, the number of microglia starts to decline and gradually decreases. During this process, M2-microglia secrete CD206, Arg, CCL22, IL-10, TGF-β, VEGF, TNF-β and BDNF. Within 24 h after the occurrence of ischemic stroke, M1-microglia begin to appear and gradually increase. M1-microglia reach their peak number 14 days after the stroke and become the predominant glial cell type. Activated astrocytes secrete IL-1β, IL-6, iNOS, TNF-α, NF-κB and MCP-1. During the acute phase, astrocytes secrete GSH, ROS, glutamate, IL-6, TNF-α and LCN2. Astrocytes also secrete laminin, fibronectin and chondroitin, which contribute to the formation of glial scars. In the subacute phase, astrocytes secrete TSP, Shh and Ca^2+^. Note: Note: LPS: Lipopolysaccharides; VEGF: Vascular Endothelial Growth Factor; TNF: Tumor Necrosis Factor; BDNF: Brain Derived Neurotrophic Factor; MCP-1: Monocyte Chemotactic Protein-1; GSH: Glutathione; ROS: Reactive Oxygen Species; LCN2: Lipocalin-2; TSP: Thrombospondins; TSP: Thrombospondins; Shh: Sonic hedgehog

Ischemic stroke is typically divided into three distinct phases: the acute phase (lasting from hours to days), the subacute phase (spanning from days to two weeks), and the chronic phase (ranging from weeks to months). During the acute phase, microglia rapidly respond and become activated, exhibiting pro-inflammatory M1 characteristics [19]. Activated M1 microglia secrete pro-inflammatory cytokines (such as IL-1β, TNF-α, ROS, and iNOS), activate immune cells, and exert phagocytic functions to clear damaged tissues and cellular debris [20]. Although these processes help remove the damage, excessive inflammatory responses can lead to neuroinflammation, neurodegeneration, and reduced neuroplasticity. Prolonged inflammation may also cause neuronal damage, dysregulation of nervous system functions, and permanent loss of neural capabilities. Following ischemic stroke, M2 microglia, which have anti-inflammatory effects, begin to appear and gradually increase in number [21]. In the later stages of the acute phase and during the subacute phase, M2 microglia gradually become dominant in the ischemic core region [21–23]. They promote tissue repair and recovery of neurological function through various means, such as promoting neurogenesis, angiogenesis, and inhibiting inflammatory responses. (Fig. 1.)

Recent studies utilizing scRNA-seq have indicated that the activation states of microglia are not merely a binary classification (M1 and M2), but rather a complex, continuous spectrum [24, 25]. This spectrum model reflects the dynamic responsiveness of microglia to a variety of stimuli and environmental factors. Specifically, in response to different types of stimuli, such as cytokines, damage-associated molecular patterns (DAMPs), neuronal activity, and the functional status of the nervous system, microglia can exhibit a range of functional characteristics [24, 26, 27]. Within this spectrum model, microglia are not only capable of displaying multiple functional states simultaneously but can also adjust their activation states in accordance with changes in the environment [26, 28]. This paradigm shifts traditional binary concept, transforming the activation of microglia into a diverse, continuous, and complex dynamic process. Given that research on the spectrum model remains incomplete, this article continues to employ the traditional binary classification (M1 and M2) to elucidate the roles of microglia and astrocytes following ischemic stroke.

## Dynamic astrocyte activation after ischemic stroke

Astrocytes are a crucial component of the CNS, playing a vital role in maintaining normal CNS physiological functions as well as responding to pathological processes such as injury and inflammation. Under physiological conditions, astrocytes maintain CNS homeostasis through four primary mechanisms: regulating ion and pH balance within the CNS; maintaining water balance both inside and outside cells; sustaining the homeostasis of reactive oxygen species (ROS); and maintaining neurotransmitter homeostasis [29–36]. Under pathological conditions, astrocytes participate in pathological responses by limiting the spread of damage, secreting cytokines, and activating signaling pathways.

To gain a comprehensive understanding of the roles that astrocytes play in different stages of ischemic stroke, researchers conducted a thorough literature search on PubMed for studies published from 2013 to the present. The search employed keywords including “Astrocyte,” “Ischemic stroke,” “Activation,” “Inflammation” and “CNS”. Additionally, researchers manually reviewed reference lists to identify other potential studies.

After an ischemic stroke, damaged tissues release inflammatory mediators, cytokines, and ROS that promote the activation of astrocytes [37]. These cytokines bind to astrocytes, further driving their activation [38]. In addition to external factors, the imbalance of intracellular calcium homeostasis and the accumulation of oxidative substances caused by ischemia also trigger astrocyte activation, activating signaling pathways such as JAK/STAT3 [37]. Astrocytes play distinct roles in different stages of ischemic stroke. The injury after ischemic stroke is split into three phases, the acute phase (0-2days in MCAO model), the sub-acute phase (2–14 days in MCAO model), the chronic phase (> 14 days in MCAO model) [39, 40]. **(**Fig. 1.**)** During the acute phase, the number of A2 astrocytes increases and reaches a peak [41]. They secrete anti-inflammatory factors to reduce inflammation and promote tissue repair. In contrast, A1 astrocytes gradually increase in the middle and late stages of the acute phase and peak in the subacute phase [41]. They release harmful factors such as iNOS and IL-1β, leading to tissue damage and exacerbated inflammation.

In the acute phase, ischemia causes massive cells death and induce inflammation. This acute injury leads to the production of ROS, which cause inflammation, neuron necrosis, and tissue damage. Activated astrocytes can secrete glutathione (GSH) to counteract the detrimental effects of ROS [42–44]. Ischemic stroke can induce the massive release of glutamate through the reversed activation of excitatory amino acid receptors-1/2 (EAAR-1/2) and the activation of volume-sensitive outwardly rectifying anion channels (VRACs) [45–47]. This leads to neuronal death [48]. Physiologically, neurons predominantly use adenosine triphosphate (ATP) to provide energy through oxidative metabolism, while astrocytes use pyruvate and lactate through glycolysis [5, 49]. After ischemic stroke, oxidative phosphorylation is restricted, leading to neuronal hypoxia-ischemia. However, astrocytes can still produce pyruvate and lactate. Lactate can then be transported from astrocytes to neurons via monocarboxylate transporters (MCTs), providing energy to neurons during the acute phase to protect them [50, 51]. Induced by multiple cytokines, astrocytes transform into reactive astrocytes. These reactive astrocytes then move to the margin of the infarct region and proliferate. They secrete extracellular matrix proteins, such as laminin, fibronectin, and chondroitin sulfate proteoglycans, to form the scar-border [52, 53]. The scar serves a dual function: it protects against the expansion of damage while also potentially impeding axonal growth and contributing to tissue damage [48]. **(**Fig. 1.**)**

In the sub-acute phase, neuroblasts from subventricular zone proliferate and migrate towards the area surrounding the infarct, eventually maturing into neurons [54]. The astrocytes release Ca^2+^, which activates Notch pathway of the neuroblast. Neuroblasts activated by Ca^2+^ require proliferation and migration abilities, and astrocytes can thus promote neurogenesis [55]. Following ischemic stroke, the dendritic spines of neurons are decreased. Some factors secreted by astrocytes are beneficial for synaptic recovery and synaptic plasticity [56, 57]. Thrombospondins (TSP) secreted by astrocytes is highly up-regulated. In MCAO models in which TSP gene had been knocked out, the recovery of synapses is restricted [48, 58]. Astrocytes also participate in angiogenesis and BBB repair. They release Sonic hedgehog (Shh) which activates RhoA/ROCK pathway in the MCAO model to promote repair [59, 60]. **(**Fig. 1**)**

Ischemic stroke can lead to cognitive impairment in chronic phase. Studies show that P2Y purinoceptor 1 (P2Y1) is the receptor involved in the release of proinflammatory cytokines [61]. Proinflammatory cytokines leads to cognitive impairment after stroke. Studies in the MCAO model show that eliminating P2Y1 in vitro gene can improve the cognitive impairment [62].

Activated astrocyte-derived neurotrophic factors, including glial cell line-derived neurotrophic factor (GDNF), nerve growth factor (NGF), and brain-derived neurotrophic factor (BDNF), play a crucial role in modulating the activity of microglia and neurons [63]. These factors not only inhibit the release of pro-inflammatory cytokines from microglia but also induce their transformation into the anti-inflammatory M2 phenotype, thereby alleviating neuroinflammation [64]. Additionally, they enhance the antioxidant capacity of neurons and promote neuronal functional recovery, providing significant support for neuroprotection following stroke.

In recent years, as research has progressed, activated astrocytes are no longer simply divided using a binary classification. The activation of astrocytes is considered a complex, continuous spectrum involving multiple subtypes and functional states [65, 66]. For example, they can be categorized based on their function (pro-inflammatory type, anti-inflammatory type), molecular markers on their surface, or different pathological phases (acute phase, sub-acute phase, chronic phase) [65–68]. Researchers have provided a detailed description of the functions of astrocytes at different pathological stages.

## The potential effect of microglia on astrocyte in ischemic stroke

Following ischemic stroke, activated microglia can influence astrocyte behavior through various pathways, thereby altering their activity and function. To gain a comprehensive understanding of the effects of microglia on astrocytes and their interactions following ischemic stroke, researchers conducted a literature search on PubMed for studies published from 2012 to the present. The search employed keywords including “Microglia,” “Astrocyte,” “Ischemic stroke,” “Inflammation,” and “Crosstalk.” After reviewing a vast array of publications, researchers selected nine relevant studies and categorized the mechanisms of microglial effects on astrocytes into the following areas: extracellular vesicles (EVs), intercellular interactions, and functions related to molecular mechanisms. The following sections will analyze these studies and provide a summarized overview to elucidate the role of microglia in modulating astrocyte behavior post-ischemic stroke.

### The functions of Microglia-EVs to the astrocytes after ischemic stroke

The BBB is crucial for maintaining the internal homeostasis of the CNS, ensuring the balance of ions, hormones, and neurotransmitters within the brain [69]. Astrocytes, as key components of BBB, play a vital role in neurotransmitter metabolism and potassium homeostasis [69, 70]. Ischemic stroke not only disrupts BBB but also exacerbates inflammatory responses. The mechanisms underlying these processes are highly complex and multifaceted, with microglia derived extracellular vesicles (microglia-EVs) exerting a particularly significant impact on astrocytes [71–73]. In the following section, researchers will provide a comprehensive summary and elucidate the impact of microglia-derived EVs on BBB and its principal constituents, particularly astrocytes, in the context of ischemic stroke.

**Fig. 2:**
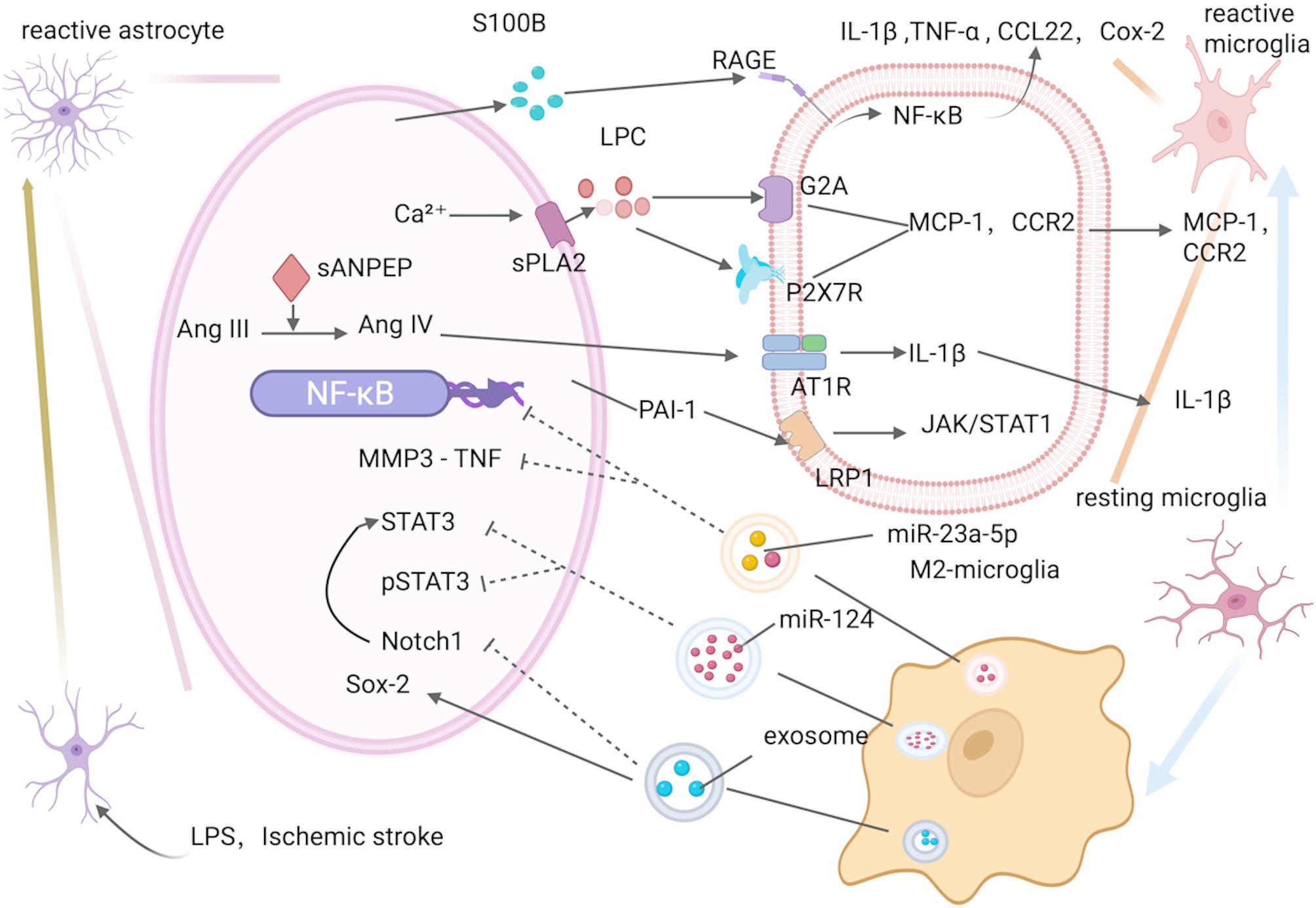
The mechanisms of crosstalk between astrocytes and microglia including EVs and molecular. 1) Activated astrocytes release S100B, which acts on RAGE on microglia, prompting the release of TNF-α, IL-1β, CCL22, and COX-2 from microglia. 2) sPLA2-A on astrocytes produces LPC, which acts on the G2A and P2 × 7R receptors on the microglial membrane, promoting the release of MCP-1 and CCR-2 from microglia. 3) Activated astrocytes produce sANPEP, which can convert Ang III to Ang IV. Ang IV acts on AT1R on activated microglia and prompts the release of IL-1β. 4) M2-microglia release extracellular vesicles rich in miR-23a-5p, which inhibits TNF in astrocytes, thereby suppressing MMPs. Additionally, miR-23a-5p inhibits the NF-κB pathway in astrocytes. 5) Extracellular vesicles released by M2-microglia contain miR-124, which can inhibit the STAT3 and pSTAT3 pathways in astrocytes. 6) Extracellular vesicles derived from microglia can also inhibit the Notch1 and promote Sox-2 pathways in astrocytes. 7) Astrocytes secrete PAI-1 to bind to LRP1, LRP1 can activate JAK/STAT1 pathway. Note: S100B: S100 calcium-binding protein B; RAGE: Receptor for Advanced Glycation End Products; COX: Cyclooxygenase; LPC: Lysophosphatidylcholine; G2A: G protein-coupled receptor 132; P2 × 7R: P2X Purinoceptor 7; MCP: Monocyte Chemotactic Protein; CCR: C-C Chemokine Receptor; sANPEP: Soluble Aminopeptidase N; MMP3: Matrix Metalloproteinase 3; STAT: Signal Transducers and Activators of Transcription; PAI-1: Plasminogen activator Inhibitor Type 1; LRP1: Low Density Lipoprotein Receptor-Related Protein 1

To explore the mechanisms by which M2 microglia-derived extracellular vesicles (M2-EVs) affect BBB and astrocytes after ischemic stroke, Pan et al. injected M2-EVs into MCAO model mice [74]. Researchers first isolated EVs from IL-4–polarized M2 microglia, labeled them with PKH26, and delivered them via tail-vein injection to MCAO mice to map their cellular uptake and distribution throughout the brain [74]. Researchers then established an in vitro oxygen–glucose-deprivation/reperfusion model to elucidate how M2-EVs, via the miR-23a-5p/TNF-MMP3-NF-κB axis, orchestrate astrocyte–endothelial crosstalk and preserve blood–brain barrier integrity [74]. Results showed that M2-EVs protected BBB integrity, reduced astrocyte death, and lessened ischemic brain damage [74]. Further research found that M2-EVs upregulated miR-23a-5p, which inhibited TNF and matrix metalloproteinase 3 (MMP3) expression to reduce inflammation and BBB disruption [74]. Similarly, Song et al. demonstrated that M2-EVs significantly increased anti-inflammatory cytokines (e.g., IL-10, TGF-β) while decreasing pro-inflammatory cytokines (e.g., TNF-α, IL-1β) [75]. Further investigation revealed that M2-EVs’ miR-124 targeted and inhibited Signal Transducers and Activators of Transcription3 (STAT3) expression [75–77]. miR-124 curbed the formation of glial scars and astrocyte proliferation by suppressing the levels of STAT3 and phosphorylated STAT3 (p-STAT3) [75–78]. **(**Fig. 2., Table 1.**)**

**Table 1:** The study evaluating interactions among microglia and astrocyte following stroke

Authors (Ref.)	Action cells	Reci Cells	Study Models	Primary Function Among the Two Glial Cells	Related Main Mechanisms
Murata et al. [91]	Microglia	Astrocyte	MCAO	Microglia increase AQP4 expression on astrocyte	AQP4
Jin et al. [169]	Microglia	Astrocyte	MCAO	Microglia limit inflammation and inhibit astrocyte	Microglia deletion
Li et al. [77]	Microglia	Astrocyte	MCAO	miR-124 reduce glial scar	EVs, miR-124/STAT3
Xin et al. [92]	Microglia	Astrocyte	OGD	M2-EVs reduce AQP4 polarization	EVs, AQP4
Liddelow et al. [93]	Microglia	Astrocyte	Cell	LPS-activated microglia release IL-1α, TNF-α, C1q to induce A1	TLR4, IL-1, TNF, C1q
Norden et al. [161]	Microglia	Astrocyte	Cell	Microglia release IL-10 to promote TNF-β releasing from astrocyte	IL-10, TNF-β
Pan et al. [74]	Microglia	Astrocyte	MCAO	M2 EVs protect the integrity of the BBB	EVs miR-23a-5p
Iizumi et al. [96]	Microglia	Astrocyte	Nrf2 knockout mice	Microglia activate Keap1/Nrf2 to enhance PPP in the astrocyte	Keap1/Nrf2, PPP, ROS, NO
Casamassa et al. [106]	Microglia	Astrocyte	Cell culture MCAO	Microglia regulate astrocyte transformation by NCX1 and Ascl1	NCX1, Ascl1
Jiang et al. [79]	Microglia	Astrocyte	BBB model	M1 EVs inhibit Notch1 and Sox2	M1-derived EVs
John et al. [107]	Microglia	Astrocyte	Cell culture	Microglia secret IL-1β to activate Rho-ROCK in astrocyte	IL-1β, Rho-ROCK
Peng et al. [119]	Astrocyte	Microglia	MCAO	Astrocyte release PRDX6‑iPLA2 to promote microglia secreting ROS	PRDX6‑iPLA2
Olschewski et al. [120]	Astrocyte	Microglia	Cell culture	RASS promotes inflammation	RAAS
Kim et al. [121]	Astrocyte	Microglia	Cell culture	ANPEP on the astrocyte promote microglia releasing IL-1β	sANPEP
Schipke et al. [123]	Astrocyte	Microglia	Cell culture	Ca²⁺ waves in astrocyte activate microglia	Ca^2+^
Orellana et al. [113]	Astrocyte	Microglia	Cell culture	Astrocyte inhibits Ca^2+^	PGE2, EP1 R, ATP, TNF-β, PanX1 Channel, Ca^2+^
Inose et al. [124]	Astrocyte	Microglia	Cell culture	LPC activates microglia releasing MCP-1, CCR-2	MCP-1, CCR2, LPC
Bianchi et al. [139]	Astrocyte	Microglia	Cell culture	S100B promotes microglia releasing TNFα, IL-1β, CCL22, COX-2	S100B
Jo et al. [143]	Astrocyte	Microglia	Mice inflammation, cell culture	ORM-2 inhibits TNFα, IL-1β releasing from microglia	ORM-2
Jeon et al. [144]	Astrocyte	Microglia	Mice model, cell culture	Not report	PAI-1
Min et al. [151]	Astrocyte	Microglia	Cell culture	Astrocyte inhibits microglia releasing HO-1	HO-1

Note: Reci: Recipient; MCAO: Middle Cerebral Artery Occlusion; AQP4: Aquaporin 4; OGD: Oxygen and Glucose Deprivation; EV: Extracellular Vesicular; AQP4: Aquaporin 4; LPS: Lipopolysaccharides; A1: Type-1 astrocyte; TLR: Toll-like Receptor; TNF: Tumor Necrosis Factor; PPP: Pentose Phosphate Pathway; ROS: Reactive Oxygen Species; BBB: Blood-Brain Barrier; PRDX6: Peroxiredoxin 6; iPLA2: Calcium-independent Phospholipase A2; RAAS: The Renin–Angiotensin–Aldosterone System; sANPEP: Soluble Aminopeptidase N; PGE: Prostaglandin E; MCP: Monocyte Chemotactic Protein; CCR: C-C Chemokine Receptor; LPC: Lysophosphatidylcholine; S100B: S100 calcium-binding protein B; CCL 22: chemokine C-C motif ligand 22; COX: Cyclooxygenase; ORM-2: Orosomucoid-2; PAI-1: Plasminogen activator Inhibitor Type 1; HO-1: Heme Oxygenase-1

Conversely, Jiang et al.‘s research has uncovered that M1-EVs (LPS-induced M1 polarization of BV2 cells and exosome isolation) can disrupt BBB and significantly increase the expression of pro-inflammatory factors in astrocytes, such as TNF-α, IL-1β, and MMP-9 [79]. Additionally, M1-EVs exacerbate the damage caused by ischemic stroke by carrying pro-inflammatory miRNAs, including miR-155 and miR-146a [79].

In summary, in ischemic stroke, M2-EVs can exert neuroprotective effects by delivering specific miRNA and regulating cytokines. In contrast, M1-EVs exacerbate post-stroke damage by delivering pro-inflammatory miRNA. This finding provides new directions and ideas for clinical treatment and drug development, that is, by utilizing protective miRNA and EVs derived from M2, it is possible to improve the prognosis of ischemic stroke. While the aforementioned studies are still based on the traditional binary classification of microglia, recent research suggests that microglial activation is a complex, continuous process that cannot be fully captured by a simple binary categorization. Therefore, the more nuanced roles of microglia remain to be further explored.

### The impact of microglia on AQP4 expression in astrocytes following ischemic stroke

**Fig. 3:**
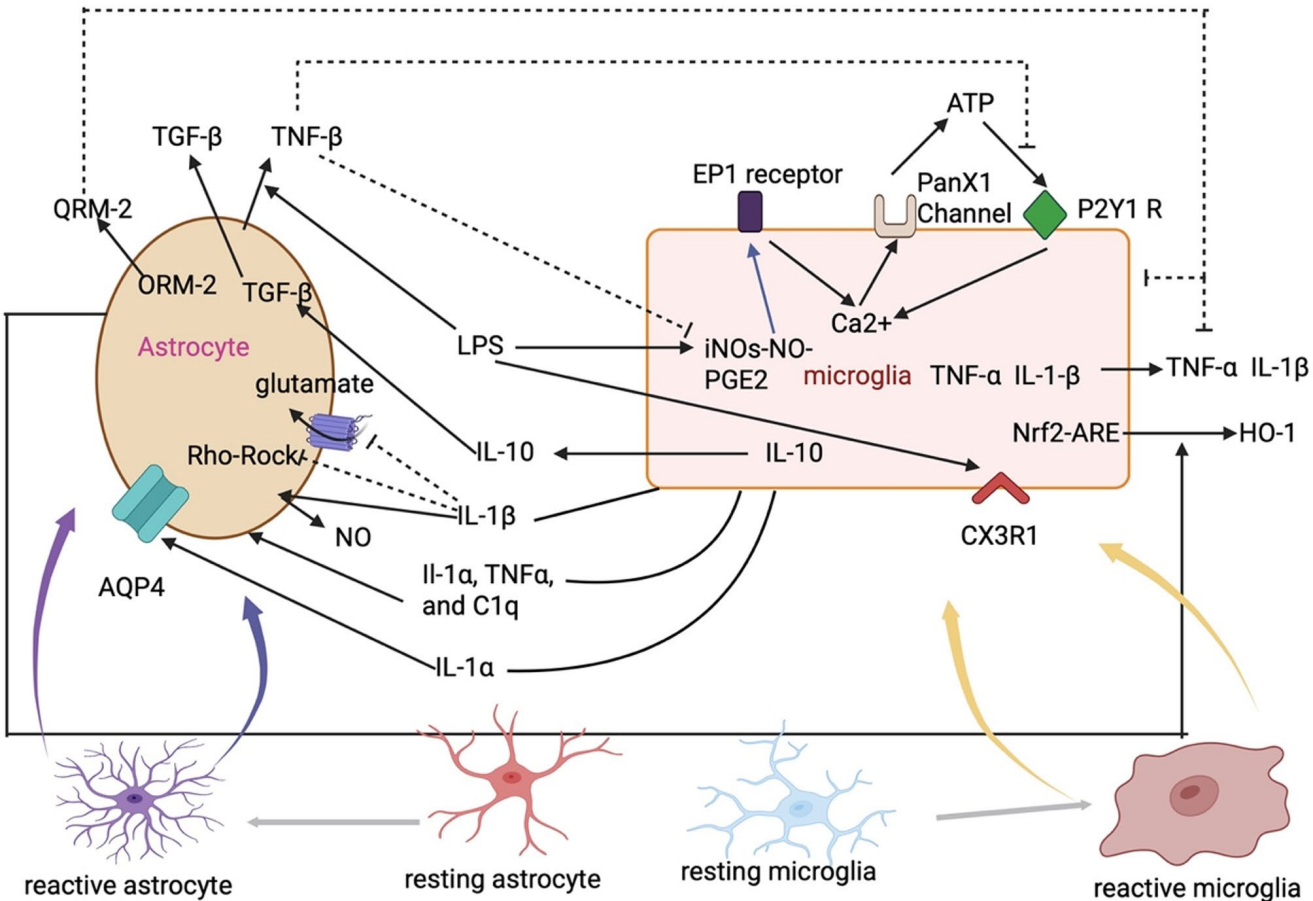
The mechanisms of crosstalk between microglia and astrocytes. 1) LPS activates microglia and produces PGE2 through the iNOS-NO pathway. PGE2 acts on the EP1 receptor, and the activation of the EP1 receptor causes the endoplasmic reticulum to release Ca^2+^, increasing intracellular Ca^2+^ levels. The increased Ca^2+^ then acts on the PanX1 channel, causing ATP to be released from microglia. ATP, in turn, acts on the P2Y1R, further increasing the Ca^2+^ concentration in microglia. 2) LPS can promote the release of TNF-β by astrocytes. TNF-β can inhibit the production of NO in microglia and suppress the P2Y1R. 3) LPS can promote the generation of IL-10 in microglia. IL-10 can act on astrocytes and promote the production of TGF-β. 4) Microglia secrete IL-1α, which acts on astrocytes, increasing the expression of AQP4 on their surface. 5) Microglia can release IL-1β, which inhibits astrocytes from taking up glutamate. IL-1β can also inhibit the Rho-Rock pathway in astrocytes. 6) ORM-2 released by astrocytes inhibits microglia activity and suppresses the release of TNF-α and IL-1β from microglia. Astrocytes promote the release of HO-1 in microglia. 7) Activated microglia secret IL-1α, TNF-α and C1q to induce astrocytes. Note: PGE2: Prostaglandin E 2; P2Y1: P2Y purinoceptor 1; AQP4: Aquaporin 4; ORM-2: Orosomucoid-2; HO-1: Heme Oxygenase-1 Note: PGE2: Prostaglandin E 2; P2Y1: P2Y purinoceptor 1; AQP4: Aquaporin 4; ORM-2: Orosomucoid-2; HO-1: Heme Oxygenase-1

Brain edema is a key factor contributing to mortality following ischemic stroke [1, 80]. It is primarily categorized into two types: cytotoxic edema and vasogenic edema [81]. Cytotoxic edema predominantly involves the swelling of astrocytes, which is largely mediated by aquaporin-4 (AQP4), a protein highly expressed in these cells [82, 83]. After ischemic stroke, microglia-derived macrophage-like cells (MG-MΦ) accumulate in the ischemic core [84–86]. Murata et al. investigated the impact of MG-MΦ on AQP4 expression in astrocytes. Previous studies have demonstrated that AQP4 expression is associated with the development of brain edema following ischemic stroke [87, 88]. Additionally, research indicates that reduced brain edema and smaller infarct volumes correlate with suppressed AQP4 expression [89, 90]. Murata et al. revealed that IL-1α significantly enhanced AQP4 expression in vitro [91]. IL-1α secreted by microglia leads to an upregulation of AQP4 in astrocytes, resulting in brain edema. (Fig. 3., Table 1.) Apart from the aforementioned mechanisms, Xin et al. also reported that the use of ischemia-hypoxia preconditioned M2-microglia derived EVs can reduce AQP4 polarization, decrease aggregation of AQP4 and decrease the levels of pro-inflammatory cytokines [92]. (Table 1.)

### The other functions and related molecular mechanism of microglia to the astrocytes after ischemic stroke

Liddelow et al. explored the specific cytokine mechanisms by which microglia activate A1-astrocytes following ischemic stroke. They demonstrated that activated microglia induce A1 astrocytes through release of IL-1α, TNF-α, and Complement Component 1q (C1q) [93]. (Fig. 3., Table 1.) In addition to cytokine release, microglia can produce nitric oxide (NO) in response to stimuli such as lipopolysaccharide (LPS) [94, 95]. Within astrocytes, the pentose phosphate pathway is a metabolic pathway that generates nicotinamide adenine dinucleotide phosphate (NADPH), which is crucial for protecting cells from oxidative stress and for biosynthetic reactions [95–97]. Keap1/Nrf2 is a critical regulator to resist oxidative reactions. Kelch-like ECH-associated protein 1 (Keap1) negatively regulates nuclear factor erythroid 2-related factor 2 (Nrf2) by targeting it for degradation under unstressed conditions [96]. Nrf2 is activated to induces antioxidant proteins, including those involved in the pentose-phosphate pathway [98–100]. Iizumi et al. reported that NO from microglia may trigger signaling pathways that activate Keap1/Nrf2 system. This activation, in turn, may enhance the pentose-phosphate pathway in astrocytes, providing them with better tools to manage oxidative stress and support neuronal survival and function [96]. (Table 1.)

Ion channels are also involved in the post-ischemic stroke. Casamassa et al. studied the effect of Sodium-Calcium Exchanger 1 (NCX1) and Achaete-Scute Complex Homolog 1 (Ascl1) in the crosstalk between microglia and astrocytes. NCX1 is crucial in maintaining calcium homeostasis in cells [101]. After ischemia, it regulates intracellular calcium levels, thereby influencing the reprogramming of astrocytes and modulating calcium-dependent signaling pathways that drive the gene expression changes necessary for astrocyte transdifferentiation [101–103]. Ascl1 is a transcription factor involved in neurogenesis [104]. In the ischemic brain, Ascl1 expression is upregulated, potentially driven by microglial signaling. It directly steers astrocytes toward a neuronal fate by activating neuronal-specific transcriptional networks [104, 105]. Activated microglia can secrete growth factors which can create a microenvironment conducive to cellular reprogramming following ischemia stroke. Microglial activation also affects the expression and activity of NCX1 and Ascl1, thereby modulating the transformation process [106]. Although Casamassa et al. elucidated the roles of NCX1 and Ascl1 in microglia-dependent astrocytic transformation, the specific mechanisms and functions still require further exploration. (Table 1.)

After the ischemic stroke, the microglia secrete IL-1β which can promote astrocytic changes and functions [107]. In their study, John et al. demonstrated that, within the context of inflammation, IL-1β triggers reorganization of actin cytoskeleton in astrocytes. This process leads to the loss of stress fibers and the localization of F-actin at the cell membrane [107]. Additionally, John et al. found that the migration ability of astrocytes treated with IL-1β is limited [107]. The underlying mechanism involves the Rho-Rock signaling pathway. IL-1β activates this pathway, which in turn phosphorylates myosin light chain (MLC), thereby controlling myosin contraction and stress fiber formation [108]. However, John et al. also revealed that IL-1β can deactivate the Rho-Rock pathway. This deactivation disrupts the interactions between F-actin and focal adhesions, as well as with Ezrin–Radixin–Moesin (ERM) proteins, ultimately inhibiting astrocyte migration [107]. Furthermore, after ischemic stroke, activated microglia release IL-1β, which can induce astrocytes to produce NO, thereby mediating neuro-degeneration [109]. (Fig. 3., Table 1.)

Microglia activated by LPS can release IL-10 [110]. IL-10 is demonstrated to play anti-inflammatory and protective roles in ischemic stroke [111]. TGF-β plays a neuroprotective and anti-inflammatory role in CNS inflammation. TGF-β mitigates inflammation by curbing microglial activation and suppressing the release of pro-inflammatory cytokines (such as IL-1β, IL-6, IL-10, TNF) and NO [112, 113]. TGF-β in activated microglia upregulates C-X3-C Motif Chemokine Receptor 1 (CX3CR1) expression and downregulates IL-1β production which can inhibit the activation of microglia [110, 114]. Norden et al. showed that during inflammation, LPS-stimulated microglia prompt astrocytes to decrease pro-inflammatory factor expression and increase TGF-β levels through IL-10 production. (Fig. 3., Table 1.)

This section provides a comprehensive overview of the impact of microglia on astrocytes following ischemic stroke. Microglia modulate astrocyte activity and their pathological responses after stroke through multiple mechanisms. On one hand, microglia secrete EVs that regulate astrocyte responses. On the other hand, they can also exert their effects by modulating AQP4 on astrocytes. In addition, microglia influence astrocyte function and participate in post-stroke pathology by activating signaling pathways such as Keap1/Nrf2, NCX1/Ascl1, Rho-Rock, and TGF-β. Although some of these mechanisms are not yet fully elucidated, these sites of action and related pathways are undoubtedly key areas for future research. For example, research on EVs and AQP4 is relatively advanced and holds promise for translation into clinical treatment and drug development. Research on EVs as carriers transporting miRNA and AQP, has been extensively reported. These directions have the potential to provide more efficient and diverse therapeutic approaches for future disease management. However, it should be noted that many of the aforementioned studies still classify activated astrocytes and microglia using the traditional binary approach. This classification method somewhat restricts the depth and breadth of the research, leading to limitations in some of the study conclusions. Therefore, a more in-depth exploration and investigation are still needed to elucidate the more specific and scientific mechanisms of action of astrocytes and microglia in pathological processes, in the hope of breaking through the limitations of the current classification and achieving a more comprehensive and precise understanding.

## The effect of astrocyte on microglia in ischemic stroke

Following ischemic stroke, astrocytes modulate the functions of microglia through various mechanisms. The crosstalk between astrocytes and microglia plays a crucial role in tissue repair, neuroprotection, and other processes after ischemic stroke.

### The effects of astrocyte-derived PRDX6-iPLA2 on microglia after ischemic stroke

Peroxiredoxin 6 (PRDX6) is composed with glutathione peroxidase and Calcium-independent Phospholipase A2 (iPLA2), which plays dual functions [115]. Previous studies reported that PRDX6 mainly express in astrocyte [116]. Chatterjee et al. discovered that PRDX6-iPLA2 can activate NADPH oxidase, which is closely associated with the production of ROS [117, 118]. Peng et al. also confirmed that ischemic stroke can activate PRDX6-iPLA2, which in turn mediates brain damage [119]. Further research has shown that after ischemic stroke, PRDX6-iPLA2 produced by astrocytes can activate NADPH oxidase 2 (NOX2) pathway and microglia, thereby promoting the generation of ROS and driving inflammatory response [119]. (Table 1.)

### The functions of RAAS system in microglia-astrocytes crosstalk after ischemic stroke

The Renin–Angiotensin–Aldosterone System (RAAS) influences astrocyte-microglia interactions during post-stroke inflammation. Angiotensin receptors 1 (AT1) and 2 (AT2), mainly on astrocytes, can be blocked by telmisartan (an AT1 inhibitor) and PD 123,319 (an AT2 inhibitor) [120]. Olschewski et al. demonstrated that telmisartan treatment in astrocyte-microglia co-cultures enhances astrocyte survival, reduces microglial iNOS, and increases IL-10 expression [120]. Research shows that telmisartan also decreases astrocyte glutamate secretion, promoting an anti-inflammatory microglial phenotype, while AT2 inhibition has the opposite effect [120]. (Table 1.) Kim et al. investigated Soluble Aminopeptidase N (sANPEP), which converts Ang III (Angiotensin III) to Ang IV in neuroinflammatory settings [121]. Astrocytes secrete sANPEP, which then converts Ang III into Ang IV [121]. Astrocytes secrete sANPEP, converting Ang III to Ang IV, which can bind to the anti-inflammatory AT4R receptor and, at high levels, to the pro-inflammatory AT1R receptor [122]. After inflammation, AT1R is highly expressed in activated microglia [121]. Kim et al. showed that Ang IV binding to AT1R mediates IL-1β release from microglia [121]. (Fig. 2., Table 1)

In summary, research indicates that astrocytes can modulate the phenotype and function of microglia through various mechanisms. On one hand, by secreting glutamate, astrocytes can promote the transformation of microglia toward the anti-inflammatory M2 phenotype. On the other hand, astrocytes secrete sANPEP, which converts Ang III into Ang IV. Ang IV, when bound to the pro-inflammatory receptor AT1R, can mediate the release of IL-1β from microglia, thereby exacerbating the inflammatory response.

### The effects of astrocyte-derived ca²⁺ on microglia after ischemic stroke

Astrocytes modulate neurons and microglial activation through calcium ion (Ca²⁺) waves. Schipke et al. observed the activity of astrocytes and microglia using calcium imaging technology [123]. The experiments of Schipke et al. found that Ca²⁺ waves can activate neighboring microglia [123]. (Table 1.) After ischemic stroke, a penumbra forms around the fresh infarct area, where microglia become highly activated [124]. Previous studies have shown that in the infarct region, Secretory Phospholipase A2 (sPLA2), a Ca²⁺-dependent enzyme from neurons and astrocytes, can stimulate microglia activation [124, 125]. X group in sPLA2 can effectively produce and release Lysophosphatidylcholine (LPC) [126, 127]. LPC has pro-inflammatory functions by attaching G protein-coupled receptor 132 (G2A) and P2X Purinoceptor 7 (P2 × 7R) [128, 129]. Inose et al. investigated whether LPC produced by sPLA2-X on astrocytes would activate microglia and promote inflammatory responses [124]. The experiment demonstrated that sPLA2 derived from astrocytes produces LPC and releases it into extracellular space, where LPC binds to receptors G2A and P2 × 7R on microglia. This leads to the upregulation of Monocyte Chemotactic Protein-1 (MCP-1) and C-C Chemokine Receptor-2 (CCR2) in microglia. Increasing evidence suggests that MCP-1 and CCR2 lead to increased vascular permeability, cell adhesion, recruitment, migration, infiltration, proliferation, cytokine release, necrosis, and phagocytosis through receptor-coupled G proteins [130, 131]. The study confirmed that astrocytes can activate microglia and promote the occurrence of inflammatory responses through the release of Ca²⁺. (Fig. 2., Table 1.)

Activated microglia have increased intracellular Ca²⁺ concentration and increased production of NO and prostaglandins, which can exacerbate inflammation and neurological damage following inflammation [132–135]. Orellana et al. found that in inflammation, LPS stimulation of microglia triggers intracellular signaling, resulting in iNOS activation, NO production, Cyclooxygenase (COX) activation, and subsequent Prostaglandin E2 (PGE2) generation [113]. Woodward et al. discovered that PGE2 can act on the Prostaglandin E2 Receptor 1 (EP1) receptor on microglia, causing the release of Ca²⁺ from the endoplasmic reticulum [136]. Ca²⁺ causes the Pannexin-1 (Pan X1) channel to open and release ATP [113]. The released ATP acts on the P2Y1 receptor, this further impacts endoplasmic reticulum, leading to the release of Ca²⁺ and intensifying inflammation [113]. However, this mechanism can be inhibited by TGF-β1 produced by astrocytes. Orellana’s experiments found that TGF-β1 secreted by astrocytes can inhibit Pan X1 channel through the iNOS-NO-COX pathway and reduce the release of ATP [113]. This can decrease the production of CO, Ca^2+^, prostaglandins, and other substances within microglia, thereby reducing inflammatory responses and protecting nervous system. (Fig. 3., Table 1)

S100 Calcium-binding Protein B (S100B), a member of the S100 protein superfamily, is predominantly expressed by astrocytes in brain and is vital for cell processes such as proliferation, differentiation, apoptosis, signaling, and metabolism [137, 138]. S100B activates microglia by binding to advanced glycation end products (RAGE), triggering release of TNF-α, IL-1β, and Chemokine (C-C motif) Ligand 22 (CCL22), and increasing COX-2 [139–142]. Bianchi et al. examined how S100B’s interaction with RAGE affects NF-κB and Activator Protein-1 (AP-1) activation and COX-2 expression [139]. Bianchi et al. determined that S100B triggers microglial activation via RAGE receptor, with NF-κB and AP-1 participating [139]. The synergistic interactions between these molecules may amplify the response of microglia under inflammatory conditions. This indicates that S100B protein released by astrocytes can activate microglia and promote inflammatory responses. (Fig. 2., Table 1)

Activated astrocytes can modulate the activity of microglia and their role in inflammatory responses through multiple mechanisms. On one hand, they can activate microglia via Ca²⁺ waves and S100B, thereby promoting the occurrence of inflammatory responses. On the other hand, TNF-β produced by astrocytes can inhibit overactivation of microglia, thereby reducing inflammatory responses.

### The effects of astrocyte-derived ORM on microglia after ischemic stroke

ORM (Orosomucoid) is categorized as immunoglobulin superfamily, and it is a type of small molecular binding protein with immune-modulating functions [143]. Jo et al. investigated the role of ORM-2 in inflammation [143]. Jo et al. discovered that ORM-2 is abundantly expressed in activated astrocytes and is secreted extracellularly [143]. ORM-2 can suppress microglial activation and decreasing secretion of pro-inflammatory cytokines like TNF-α and IL-1β [143]. The research shows that ORM-2 can suppress overactivation of NF-κB and Mitogen-Activated Protein Kinase (MAPK) pathways in microglia to alleviate the inflammatory [143]. ORM-2 provides protection to neurons, helps to maintain neural function and reduce cell damage. This study reveals the function of ORM-2 and complexity of cytokines and their signaling pathway adjustments in inflammation. This regulatory mechanism is not only related to the function of microglia but may also affect the overall inflammatory process. (Fig. 3., Table 1)

### The effects of astrocyte-derived PAI-1 on microglia after ischemic stroke

Plasminogen activator inhibitor type 1 (PAI-1) primarily inhibits both urokinase type plasminogen activators (uPA) and tissue type plasminogen activators (tPA), which are responsible for fibrinolysis [144]. PAI-1 is a major inhibitor of fibrinolysis and participate in thrombosis formation. Recent research found that PAI-1 can regulate nervous system [145, 146]. Jeon et al. explored how PAI-1 regulates the motility and phagocytic activity of microglia. Research indicated that both astrocytes and microglia can produce PAI-1, while astrocytes are the main source [147–150]. Jeon et al. discovered that PAI-1 can regulate movement and migration capabilities of microglia [144]. Jeon et al. indicated that PAI-1 inhibits phagocytic action of microglia by blocking vitronectin/ITGB 3/TLR 2 complex. In summary, astrocytes inhibit the activity of microglia by secreting PAI-1, thereby suppressing inflammatory responses. (Fig. 2., Table 1)

### The functions of astrocytes-derived HO-1 on microglia after ischemic stroke

Heme oxygenase-1 (HO-1) serves as a significant regulatory factor endowed with antioxidant and anti-inflammatory capabilities [151]. Following ischemic stroke, HO-1 can protect tissues from damage through various mechanisms, including its antioxidant and anti-inflammatory properties. Min et al. demonstrated that microglia treated with astrocyte-conditioned medium (ACM) exhibited elevated HO-1 levels [151]. Min et al. also found that ACM could trigger the nuclear translocation of (Nuclear Factor Erythroid 2-related Factor 2) Nrf2 in microglia, enhancing its binding to antioxidant response elements (ARE), which in turn lead to an increase in HO-1 expression and activity [151]. These reactions might decrease levels of intracellular ROS and inhibit production of interferon-gamma (IFN-γ), thereby curbing inflammatory responses in microglia [151]. This study reveals that astrocytes can inhibit inflammation by upregulating the expression of HO-1 in microglia [151]. (Fig. 3., Table 1)

In this section, researchers have studied the impact of astrocytes on microglia following ischemic stroke. The findings indicate that after ischemic stroke, astrocytes can activate microglia and promote inflammatory responses through the production or secretion of factors such as PRDX6, sANPEP, Ca²⁺, and S100B. Conversely, astrocytes can also produce substances like ORM-2 and PAI-1 that act on microglia to inhibit their activity and thereby curb inflammatory reactions. This intricate interplay plays a crucial role in tissue damage and repair after ischemic stroke. Inflammation caused by ischemic stroke significantly exacerbates neurological damage and severely impacts patient outcomes. Therefore, mitigating post-stroke neuroinflammatory damage has become a key therapeutic focus. Future research and treatment strategies should concentrate on inhibiting astrocyte-mediated microglial activation and enhancing astrocyte-mediated suppression of microglia to reduce inflammation by targeting key sites of action. These mechanisms not only offer new directions for clinical research but also provide important targets for drug development. For instance, based on the aforementioned mechanisms, it is possible to reduce inflammatory damage to the brain by blocking the pro-inflammatory processes of astrocytes and promoting their anti-inflammatory processes. However, current research remains immature, with many studies still relying on traditional concepts, such as the binary classification of activated microglia and astrocytes. This limits the depth and breadth of the research, and more specific and scientific mechanisms of action still need to be further explored. Therefore, future research needs to further refine these mechanisms, which also points the way for future studies.

## The emerging contribution of Astrocytes-Microglia crosstalk in neuron survival and function

This section will integrate studies from previous studies to explore the decisive impact of the crosstalk between microglia and astrocytes on neuronal fate following ischemic stroke. It will elucidate how this crosstalk contributes to either neuroprotection or neurodegeneration and provide insights into potential therapeutic strategies that may improve outcomes for stroke patients. The crosstalk between astrocytes and microglia plays a complex and crucial role in neuronal protection and survival. This crosstalk can influence neurons through a variety of mechanisms, which are outlined as follows.

### The potential effect of astrocytes on the neuron

#### The beneficial effects on the neurons

##### Metabolic and energetic supply

Following ischemic stroke, neurons in the affected area lose their normal energy supply, leading to neuronal damage and death. At this time, glycogen stored in astrocytes is broken down into glucose and astrocytes can also take up glucose from blood vessels [152, 153]. Astrocytes convert intracellular glucose into lactate and transport it outside the cell via MCT1 and MCT4 [153]. Neurons can take up lactate through MCT2 [153]. The lactate provides an alternative energy source to support the survival and functional recovery of neurons damaged by ischemia.

##### Antioxidant function

As previously mentioned, ischemic stroke activates EAAR-1/2 and VRACs, leading to the release of large amount of glutamate [46, 47]. The massive accumulation of glutamate elevates intracellular Ca^2+^ levels, causing neuronal damage and death [55]. Astrocytes can reduce the damage of glutamate to neurons by taking up and transforming glutamate.

##### Antioxidant function

Activated astrocytes secrete GSH, which mitigates ROS-induced toxicity and oxidative stress following ischemia. By reducing the levels of oxidant substances, astrocytes can protect neurons from further damage.

##### Glia Scar formation

Reactive astrocytes migrate to the margin of infarct region and secrete extracellular matrix proteins such as laminin, fibronectin and chondroitin sulfate proteoglycans to form the glia scar. The scar formation prevents the expansion of damage and protect surrounding neurons from injury.

##### Repair and neurogenesis functions

Astrocytes release Ca^2+^ to stimulate proliferation and activity of neural stem cells [55]. Additionally, astrocytes also release TSP to repair neuronal protrusions. Astrocytes are also involved in angiogenesis of damaged tissue and the repair of the BBB [58]. However, it is important to note that these experimental results were obtained from rodent models, which have a higher density of neural precursor cells. Therefore, whether these findings are applicable to humans still requires further validation.

##### Other mechanisms

Astrocytes can also protect neurons through other molecular mechanisms or via the release of extracellular vesicles. Zhang et al. indicated that Vascular Endothelial Growth Factor (VEGF) can enhance vascular formation following ischemic stroke and reduce impairment of neuronal cell function [154]. Astrocyte-derived Brain Derived Neurotrophic Factor (BDNF), Fibroblast Growth Factor (FGF), and Glial Cell Line-derived Neurotrophic Factor (GDNF) play roles in safeguarding neurons, fostering axonal growth, and supporting synaptic regeneration [155–157].

#### The detrimental effects on the neurons

After ischemic stroke, astrocytes secrete multiple pro-inflammatory cytokines to promote inflammation and contribute to neuron damage. Astrocytes release S100B, which stimulates microglia to produce TNF-α, IL-1β, CCL22, and increases COX-2 expression [21–23, 139]. Reactive astrocytes produce IL-6, TNF-α, IL-1α, IL-1β, IFN-γ, NO, all of which are harmful to the neurons. The astrocytes secrete extracellular matrix proteins to form glia scar [52]. The formation of scar inhibits neuron regeneration and axonal repair. Activated astrocytes can have a direct toxic impact on neurons through release of ROS and Reactive Nitrogen Species (RNS) [158]. Astrocytes can promote functional recovery, as well as exacerbate damage and hindrance to neurogenesis.

### The potential effect of microglia on the neurons

#### The beneficial effects on neurons

##### Clearance and engulfment

Following ischemic stroke, damaged and necrotic tissues accumulate, further triggering inflammatory responses that exacerbate destruction of neurons by inflammation. Activated microglia can phagocytose and clear these necrotic tissues and substances, reducing neurons damage and decreasing neuronal death.

##### Anti-inflammation cytokines

M2-microglia release anti-inflammatory cytokines like IL-4, Ym1/2, IL-10, TGF-β, and IL-13 to counteract inflammation [4, 15]. This helps to reduce neuronal damage and death, and protect function and survival of neurons.

##### Neurotrophic factors

M2-type microglia release neurotrophic factors including Insulin-like Growth Factor 1 (IGF-1), which prevents cell death and promotes growth and maturation of neural precursor cells [6].

#### The detrimental effects on the survival and function of neurons

##### Pro-inflammation cytokines

M1-type microglia release pro-inflammatory cytokines, such as TNFα, IL-1β, IL-6, which exacerbate inflammatory responses and cause oxidative stress [15]. This causes neuronal damage and destruction, resulting in deficits in neurological function and brain edema.

##### Pyroptosis

Microglial activation-induced pyroptosis is dependent on caspase-1/11 and formation of the (NOD-like Receptor Protein 3) NLRP3 inflammasome [159, 160]. Pyroptosis induced by microglial activation can exacerbate neuronal damage and reduce neuronal survival through these mechanisms.

### The potential effect of microglia-astrocyte interaction on the neuron

The crosstalk astrocytes-microglia crosstalk can lead to neuronal survival, regeneration, repair, as well as injury and necrosis. These effects are mediated by different mechanisms and interactions. The impact of the crosstalk between astrocytes and microglia on neurons after ischemic stroke is double-edged, capable of protecting neurons from damage as well as promoting neuronal injury and necrosis. Here, summarizing the aforementioned research, researchers list the effects of the crosstalk between astrocytes and microglia on neurons.

#### The protective effects on neurons

The crosstalk between microglia and astrocytes can impact neuronal survival through various mechanisms, such as EVs, cytokines, and neuro-inflammation. Summarizing protective effects of the EVs exchange between astrocytes and microglia on neurons as follows. Pan et al. discovered that M2 microglia’s EVs, enriched with miR-23a-5p, can suppress TNF expression in astrocytes, reducing MMP-3 formation and shielding the BBB from inflammation [74]. Protecting BBB is beneficial for neuronal survival. Li et al. found that miR-124 from M2-EVs inhibits glial scar formation [77]. Inhibiting glial scarring can prevent excessive neuronal destruction and promote neuronal and synaptic repairThe miR-124/STAT3 pathway can also convert astrocytes into neural progenitor cells. Xin et al. found that hypoxia-preconditioned M2-EVs can reduce AQP4 polarization in astrocytes and decrease pro-inflammatory cytokines, thereby protecting neurons [92].

Astrocytes and microglia can also affect inflammation after ischemic stroke by releasing and transferring cytokines to protect neurons. Through experiments, Norden et al. found that activated microglia produce IL-10 and act on astrocytes [161]. This can decrease the production of pro-inflammatory factors and stimulate the release of TGF-β. TGF-β can also acts on microglia, reducing their activity and inhibiting their pro-inflammatory state. Inhibiting occurrence and development of inflammation can reduce neuronal damage. Following ischemic stroke, the production of nitric oxide and increased Ca^2+^ levels by microglia exacerbate inflammatory response and promote occurrence of inflammation. Orellana et al. found that astrocytes produce TNF-β, which can inhibit production of NO and reduce Ca^2+^ concentration in microglia by inhibiting ATP releasing through the Pan X1 channel [113]. This, in turn, alleviates inflammation and protects neurons. Jo et al. determined that ORM-2, released by astrocytes, can suppress microglial activation and decrease the secretion of pro-inflammatory cytokines like TNF-α and IL-1β [143]. ORM-2 can reduce microglial inflammation by dampening the overactivation of pathways such as NF-κB and MAPK. Jeon et al. also found that PAI-1 secreted by astrocytes suppresses the phagocytic activity of microglia by blocking the fibronectin/ITGB3/TLR2 complex [144]. This could reduce damage to neurons and protect their functions. Astrocytes can influence microglia by regulating HO-1 expression and ROS levels, thereby preventing excessive brain inflammation [151].

#### The harmful effects on neuron

Murata et al. revealed that after ischemic stroke, IL-1α released from microglia causes an increase in AQP4 in astrocytes, leading to brain edema [91]. Cerebral edema may cause or exacerbate accumulation of excitotoxic substances, leading to excitotoxicity and neuronal damage. Hypoxia and metabolic disorders accompanying cerebral edema can lead to insufficient energy supply, affecting the metabolic activities of neurons and thereby impacting their function. Cerebral edema increases intracranial, compressing surrounding brain tissue, including neurons. This pressure can cause morphological changes in neurons and even lead to neuronal death. Peng et al. demonstrated that after ischemic stroke, PRDX6-iPLA2 generates ROS by activating the NADPH oxidase complex. ROS can exacerbate neuroinflammatory responses and lead to neuronal damage [119]. Kim et al. demonstrated that astrocytes secrete sANPEP, which then converts Ang III into Ang IV [121]. The sANPEP bind with AT1R on microglia and promote microglia releasing IL-1β [121]. The IL-1β is pro-inflammation cytokines which exacerbates inflammatory responses. Bianchi et al. found that S100B secreted by astrocytes can activate microglia and stimulate secretion of inflammatory cytokines, such as TNF-α, IL-1β, and CCL22 and upregulate expression of the pro-inflammatory enzyme COX-2 [139]. This will upregulate inflammation and cause the damage of neurons.

### The potential effect of microglia-astrocyte interaction on the other cells in CNS

In addition to their impact on neurons, the interaction between astrocytes and microglia following ischemic stroke also affects other cells in the CNS. Oligodendrocytes are key cells in the CNS for myelin formation and maintenance of homeostasis. After a stroke, activated microglia stimulate astrocytes through gap junctions and by secreting inflammatory factors such as IL-1α and TNF-α. Among these, A1 astrocytes can induce the death of oligodendrocytes [162]. However, in the later stages of stroke, microglia and astrocytes with protective functions can promote the generation of oligodendrocytes and myelin repair [163].

In the CNS, pericytes play a crucial role in maintaining vascular stability and participating in angiogenesis, and are also key to the integrity of the BBB. However, following ischemic stroke, astrocytes and microglia become mutually activated and release pro-inflammatory cytokines such as TNF-α and IL-1β [164]. These cytokines not only cause damage to and death of pericytes, but also stimulate pericytes to release more cytokines, thereby further exacerbating the inflammatory response [164]. The damaged pericytes directly affect endothelial cells, leading to disruption of the integrity of the BBB and a significant increase in its permeability [164, 165]. This change allows harmful substances to more easily enter the brain, worsening brain tissue injury [166]. However, in the mid-to-late stages of stroke, as the inflammatory response subsides, M2 microglia and astrocytes begin to exert their reparative functions [167]. The neurotrophic factors they release can promote the recovery of pericyte function, thereby aiding in the repair of the BBB and creating favorable conditions for brain recovery [167].

Similarly, in the early stages of ischemic stroke, activated microglia and astrocytes secrete inflammatory factors and chemokines (such as CCL2), as well as release EVs. These substances can cause endothelial cells to contract, leading to the destruction and death of endothelial cells, and the disruption of tight junctions between endothelial cells, ultimately compromising the integrity of the BBB [164, 168]. In the late stages of stroke, microglia and astrocytes secrete neurotrophic factors that can protect endothelial cells and promote their repair [164].

## Conclusion

In the preceding text, we systematically reviewed and summarized the individual roles of astrocytes and microglia following ischemic stroke, with a particular focus on how their interactions impact the CNS. Astrocytes and microglia play crucial roles in the nervous system. Investigating their interplay not only deepens our understanding of the pathological mechanisms underlying ischemic stroke but also uncovers the protective and detrimental mechanisms between glial cells. This knowledge is of paramount importance for gaining insights into the pathological processes of neurons and other neural cells, as well as for safeguarding the survival of neural cells. Although we have endeavored to provide a comprehensive review and summary of the relevant research, it is important to acknowledge that both the field of study and this article have certain limitations.

Firstly, the majority of current studies are still based on the traditional binary classification of astrocytes and microglia, namely M1/M2 and A1/A2. However, this classification has obvious limitations. In fact, M1, M2, A1, and A2 do not exist in isolation; they are not simply two distinct states. After ischemic stroke occurs, the activation process of astrocytes and microglia is a continuous spectrum, and there may be multiple intermediate states, that is, different layers of microglia and astrocytes may appear simultaneously. Moreover, activated microglia and astrocytes are not limited to the two phenotypes of M1, M2, A1, and A2. Existing research indicates that, depending on different classification criteria, they may have a variety of more detailed subtypes, and different subtypes play different roles in the pathophysiological process of ischemic stroke. Due to the limitations of existing research, this paper still adopts the traditional binary classification and fails to fully reflect the complex diversity of microglia and astrocytes and their interactions at different stages. Secondly, current research mainly focuses on the early and late stages after stroke, while the interactions in the intermediate stage are relatively less understood, especially the interactions during the phenotypic transformation process of M1, M2, A1, and A2. This temporal research gap limits our comprehensive understanding of the entire stroke pathological process. Furthermore, the interactions between astrocytes and microglia discussed in this paper mainly focus on cytokines, extracellular vesicles, and direct channel connections. However, with the in-depth of research, more and more evidence shows that there may be more new interaction mechanisms between these two types of cells, and these new mechanisms need to be further discovered and explored.

In summary, the interaction between astrocytes and microglia plays a crucial role after ischemic stroke. Reviewing and summarizing existing research results and analyzing their limitations will help clarify the direction for future research and find new breakthrough points for stroke-related studies.

## Data Availability

No datasets were generated or analysed during the current study.

## Abbreviations

BBB: Blood-Brain Barrier
MCAO: Middle Cerebral Artery Occlusion
CNS: Central Nervous System
NKA: Na+/K+-ATPase
SLC: Solute Carrie GABA: Gamma Aminobutyric Acid
IL: Interleukin
iNOS: Inducible Nitric Oxide Synthase
EAAT: Excitatory Amino Acid Transporters
Ym1/2: Chitinase 3-like Protein 1 and 2
AQP4: Aquaporin 4
ROS: Reactive Oxygen Species
GSH: Glutathione
VRACs: Volume-Sensitive Outwardly Rectifying Anion Channels
TGF: Transforming Growth Factor MCT: Monocarboxylate Transporters
NO: Nitric Oxide
DAMPs: Damage-Associated Molecular Patterns
C1q: Complement Component 1q
ATP: Adenosine Triphosphate
Keap1: Kelch-like ECH-associated protein 1
Nrf2: Nuclear Factor Erythroid 2-related Factor 2
TSP: Thrombospondins
P2Y1: P2Y purinoceptor 1
Shh: Sonic hedgehog
EV: Extracellular Vesicular
MMP3: Matrix Metalloproteinase 3
MLC: Myosin Light Chain
TNF: Tumor Necrosis Factor
TLR: Toll-like Receptor
STAT: Signal Transducers and Activators of Transcription
CSF: Cerebrospinal Fluid
MG-MΦ: Microglia-derived Macrophage-like Cells
CSF1R: Colony Stimulating Factor 1 Receptor
LPS: Lipopolysaccharides
NADPH: Nicotinamide Adenine Dinucleotide Phosphate
Asc1: Achaete-scute Complex Homolog 1
NCX1: Sodium-calcium exchanger 1
ERM: Ezrin–Radixin–Moesin
CX3CR1: C-X3-C Motif Chemokine Receptor 1
PRDX6: Peroxiredoxin 6
iPLA2: Calcium-independent Phospholipase A2
NOX2: NADPH oxidase 2
RAAS: The Renin–Angiotensin–Aldosterone System
AT: Angiotensin receptor
Ang: Angiotensin
EP1: Prostaglandin E2 Receptor 1
PanX1: Pannexin-1
ARB: Angiotensin Receptor Blocker
sANPEP: Soluble Aminopeptidase N
LPC: Lysophosphatidylcholine
P2X7R: P2X Purinoceptor 7
G2A: G protein-coupled receptor 132
MCP: Monocyte Chemotactic Protein
CCR: C-C Chemokine Receptor
COX: Cyclooxygenase
sPLA2: Secretory Phospholipase A2
PGE: Prostaglandin E
S100B: S100 calcium-binding protein B
RAGE: Receptor for Advanced Glycation End Products
CCL22: Chemokine (C-C motif) Ligand 22
AP-1: Activator Protein-1
ORM: Orosomucoid
MAPK: Mitogen-Activated Protein Kinase
PAI-1: Plasminogen activator Inhibitor Type 1
uPA: Urokinase type: Plasminogen Activators
tPA: Tissue type Plasminogen Activators
HO-1: Heme Oxygenase-1
ACM: Astrocyte-Conditioned Medium
ARE: Antioxidant Response Elements
IFN-γ: Interferon-Gamma
VEGF: Vascular Endothelial Growth Factor
BDNF: Brain Derived Neurotrophic Factor
FGF: Fibroblast Growth Factor
GDNF: Glial Cell Line-derived Neurotrophic Factor
RNS: Reactive Nitrogen Species
IGF-1: Insulin-like Growth Factor 1
